# Optimization of Continuous Casting for Preventing Surface Peeling Defects on Titanium-Containing Ferrite Stainless Steel

**DOI:** 10.3390/ma16041461

**Published:** 2023-02-09

**Authors:** Chien-Cheng Feng, Ming-Hong Lin, Yi-Cheng Chen, Shih-Fu Ou, Ching-Chien Huang

**Affiliations:** 1 Department of Mechanical Engineering, National Kaohsiung University of Science and Technology, Kaohsiung 807, Taiwan; 2 Department of Mold and Die Engineering, National Kaohsiung University of Science and Technology, Kaohsiung 807, Taiwan

**Keywords:** stainless steel, overheat, casting speed, Taguchi method, recycling, sustainable

## Abstract

The surfaces of cold-rolled titanium-containing ferrite stainless steel (TCFSS) strips produced from scrap are prone to severe peeling owing to cracking near slab inclusions during hot rolling. In this study, the Taguchi method was used to prevent peeling defects and clogging of the submerged entrance nozzle, and the optimal casting parameters, such as the degree of casting overheating, casting speed, stirring time, and inclination, were determined. The results showed that increasing the degree of casting overheating and decreasing the casting speed prevented clogging and effectively mitigated peeling defects. Sample A3B1C3D2 had the optimal parameters to reduce the clog thickness to less than 1.5 mm, i.e., a degree of overheating of 60 °C, a casting speed of 0.80 m/min, a stirring time of 12.0 s, and an inclination angle of 6.0°. Sample A3B1C1D3 had the optimal parameters to prevent peeling defects, i.e., a degree of overheating of 60 °C, a casting speed of 0.80 m/min, a stirring time of 10.0 s, and an inclination angle of 6.2°. When casting using these optimal parameters, no peeling defects were observed on the surfaces of the TCFSS strips. The TCFSS strips produced using the optimized parameters exhibited the required mechanical properties and satisfied the design criteria. The parameters included a tensile strength of ≥415 MPa, a yield strength of ≥205 MPa, an elongation of ≥22%, and a hardness of ≤89 HRB.

## 1. Introduction

Recycling is crucial for preserving resources such as stainless steel. In a circular economy, the production of stainless steel from scrap is a sustainable method for producing stainless steel products. The global shortage of nickel resources has severely affected the production of nickel-containing stainless steel in recent years. Therefore, stainless steel without nickel has been developed. Currently, nickel-containing stainless steel is gradually being replaced by nickel-saving stainless steel in applications such as the manufacturing of elevator panels, building decorations, heat exchangers, and automobile exhaust pipe systems. Recently, titanium has been added to steel as a stabilizing element to decrease the content of interstitial elements such as carbon and nitrogen. Titanium-containing ferrite stainless steel (TCFSS) has gained considerable attention owing to its improved mechanical properties and corrosion resistance [1]. However, cold-rolled TCFSS strips are prone to developing peeling defects on their surfaces during the rolling process.

In steelmaking, the ease of formation and precipitation of titanium nitride increases as the temperature of the molten steel decreases [2]. Consequently, the uncontrolled addition of titanium may result in the inclusion of titanium compounds in stainless steel, thereby affecting the surface quality, toughness, and ductility of the product [3]. Furthermore, the calcium/aluminum ratio should be >0.15 to improve the cleanliness of the corresponding liquid steel [4]. In continuous casting, fixing the overheating temperature of the casting at ≤35 °C prevents breakout (caused by the melting and cracking of the slab shell) [5]. Yan et al. [6] used a nozzle cooling system to control heat flux, which can increase the equiaxed crystal ratio of steel. The influence of casting speed on the surface fluctuation of molten steel during continuous casting was investigated by Zhang et al. [7]. The authors recommended that the rate at which the speed is changed should be low at high casting speeds.

In this study, recycled stainless steel scraps were used to manufacture cold-rolled stainless steel via steelmaking processes such as smelting, casting, and rolling, and the formation of surface defects in cold-rolled steel was investigated. The mechanical properties, surface quality, and microstructure of steel are significantly influenced by steelmaking processes such as casting and rolling [8]. The degree of overheating affects the probability of the formation of shrinkage cavities and pores during solidification [9]. Furthermore, if the temperature of the steel in the tundish is too low, floating into the tundish flux becomes difficult for the inclusion, and the nozzle often becomes clogged. An increase in casting speed increases production and improves steel quality. However, breakout may occur at high casting speeds owing to the thin solidified shell [10]. In previous studies, casting speeds of less than 2 m/min have been applied (for example, 0.6–1.4 m/min [7], 1.3 m/min [11], 1.6–1.8 m/min [12], and 0.7 m/min [13]).

Appropriate stirring has many benefits, including the refinement of the solidified structure, a reduction in the number of inclusions, and the improvement of the steel surface [14]. Rational argon stirring with optimized nozzle positions, the top area of the porous brick, and gas flow rate can be used to efficiently reduce oxygen content and inclusions [15]. Yue et al. [16] investigated the effects of fluid flow on the removal rate of inclusions in a tundish by comparing two stirring methods. The results indicated that an asymmetrical flow field increases the floatation rate of the inclusions. In addition, the asymmetrical flow field can be improved by rotating the inlet direction of the swirling chamber by 60°. Previous studies have focused on the effects of one or two parameters on the behavior of molten steel and steel slab quality [6,9,10,13]. However, systematic analyses of the effects of multiple parameters on the large-scale manufacturing of steel strips are presently lacking. Accordingly, the Taguchi method was used to analyze the influence of four parameters on the surface quality of cold-rolled steel. These parameters included the degree of casting overheating (i.e., the casting and solidification temperatures of molten steel), casting speed, stirring time, and inclination.

## 2. Materials and Methods

**Figure 1:**
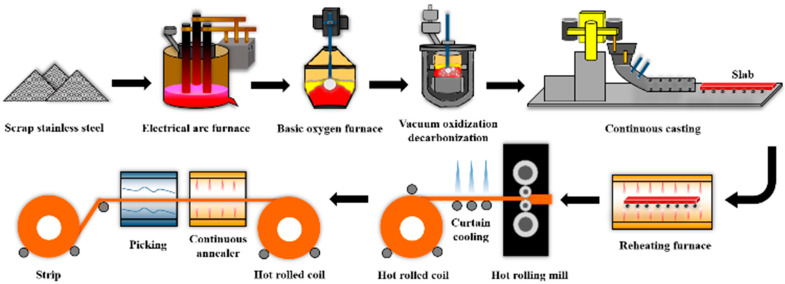
Schematic of the production process of cold-rolled TCFSS strips.

A schematic of the steelmaking process for the TCFSS strips is shown in Figure 1. Scrap stainless and alloy steel (60–63% chromium with ≤0.1% calcium) were placed in an electrical arc furnace with a carbon electrode (610 mm) to generate an arc and melt the alloy. Subsequently, oxygen gas was blown over the material to decarburize the molten steel.

The molten steel was then transferred to a basic oxygen converter to reduce the carbon content, and ferrosilicon was added to reduce the chromium content. To further reduce the carbon and nitrogen content, oxygen gas was blown into the molten steel in a vacuum oxidization decarbonization furnace (vacuum ≤1 mbar). Subsequently, 650 kg of aluminum wire (≥99.6%; D = 9.5 ± 0.5 mm), 300 kg of calcium wire (97 ± 1%; D = 9 ± 0.5 mm), and ferro-titanium ingots were added to the molten steel. Optical emission spectrometry (Model ARL 3460) indicated that the chemical composition of the TCFSS was 0.01C-17.73Cr-0.05Al-0.01Ca-0.0072N-0.61Ti. A copper mold with water cooling at 6–7 °C was used as a casting mold during the continuous casting. As the slabs left the mold, a thin solidified shell formed on their surfaces, and a water spray was applied to solidify them. The slabs with a thickness of 175 mm were first cooled to 250 °C and then subsequently heated to 1090 °C in a reheating furnace for 4 h. They were then hot rolled to obtain TCFSS strips with a thickness of 4.0 mm. The TCFSS strips were then annealed at 990 °C in a continuous annealing furnace. Next, the TCFSS strips were pickled to simulate the pickling caused by cold rolling. The yield strength, tensile strength, and elongation of the TCFSS strips were measured using a universal testing machine (ZwickRoell Z250, Ulm) at a rate of 3 mm/s. The surface of the cold-rolled TCFSS strips were etched using a mixed acid solution (1% HNO_3_ + 10% HCl + 10% H_2_O) and examined using optical microscopy (OM, OLYMPUS PMG3, Tokyo) to analyze their microstructures. The peeled samples were ground, polished, and examined using a scanning electron microscope (SEM, JEOL JSM-7000F, Tokyo). The surface composition was determined using energy-dispersive X-ray spectroscopy (EDS). The peeling defects on the surfaces of the TCFSS strips were measured using calipers (Mitutoyo, Kawasaki).

The experimental parameters designed using the Taguchi method are listed in Table 1. Four factors, namely, the degree of overheating (the casting and solidification temperatures of the molten steel), casting speed, stirring time, and inclination were each designed with three levels. Variance analysis (ANOVA) was performed to determine the influence of these factors on peeling width and clog thickness. The signal-to-noise (S/N) ratio was used to optimize the responses of the variables. In the S/N ratio, the signal and noise refer, respectively, to the real value that is desired and to undesired factors in the measured values. Three fundamental classifications can be used to decide the best outcome of an experiment. The smaller-the-better characteristic formulas used in this study are given below.

(1) $$S/N_{i}=-10\;\log_{10}\left[\frac{1}{n}\sum_{i=1}^{n}y_{i}{}^{2}\right]$$

where *n* is the number of measurements and y_i_ is the i-th observation. The unit of the S/N ratio is dB.

The total sum of squares (SS_T_) was calculated according to the equation below:

(2) $${\mathrm{SS}}_{T}=\sum_{i=1}^{n}{\left(\eta_{i}-\eta_{m}\right)}^{2}$$

where the S/N ratio for η_i_ is calculated from the measured isolation line width and η_m_ is the average value of the S/N ratio calculated from the measured isolation line width. SS_T_ represents the total sum of squares of each factor. Below, A, B, and C represent power, pulse repetition rate, and defocusing distance, respectively.

(SS_T_ = SS_A_ + SS_B_ + SS_C_).

(3) $${\mathrm{SS}}_{A}=\sum_{i=1}^{k_{A}}n_{Ai}{\left(\eta_{Ai}-\eta_{m}\right)}^{2}$$

where k_A_ is the number of levels of factor A, n_Ai_ is the number of experiments of factor A at the *i*-th level, η_Ai_ is the S/N ratio of factor A at the *i*-th level, and η_m_ is the average S/N ratio.

## 3. Results and Discussion

The peeling defects on the surfaces of the TCFSS strips were observed microscopically. Subsequently, the compositions of the clogs in the submerged entrance nozzle were analyzed to understand the formation mechanism of the surface peeling defects. The Taguchi method and ANOVA were applied to analyze the influence of the parameters on the clog thickness and peeling width, and the optimal parameter combination was determined. Finally, the mechanical properties and composition of the TCFSS strips that were fabricated using the optimal parameters were tested.

### 3.1. Surface Peeling Defects of the Cold-Rolled TCFSS Strips

**Figure 2:**
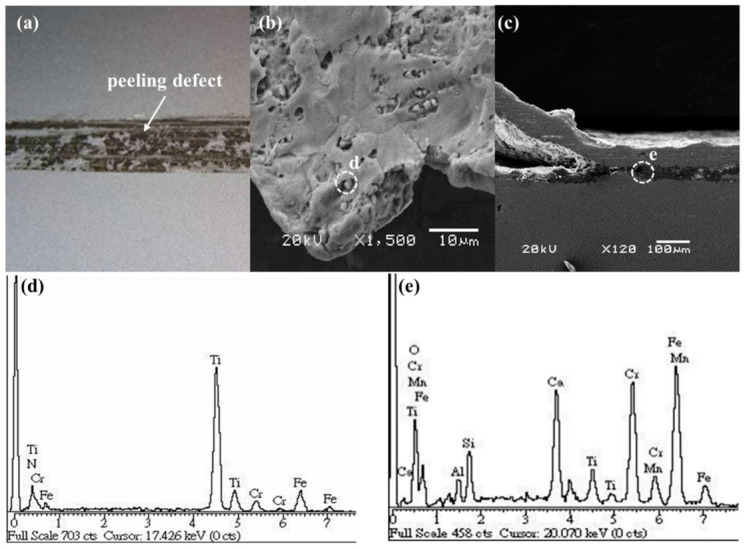
(**a**) Photograph of the cold-rolled TCFSS strip, SEM images of (**b**) the inner surface of the peeling defect and (**c**) the peeling defect present at the interlayer, and EDS profiles of (**d**) position “d” in (**b**) and (**e**) position “e” in (**c**).

As is shown in Figure 2a, the surfaces of the cold-rolled TCFSS strips exhibit distinct peeling characteristics that appear as black patches. Diamond-shaped inclusions on the inner surfaces of the peeling defects were observed during SEM analysis (Figure 2b). The EDS results in Figure 2d indicate that the diamond-shaped inclusions contain high amounts of titanium and nitrogen. Figure 2c shows a cross-sectional SEM image of a TCFSS strip and an interlayer between the peel and substrate with high titanium, aluminum, and oxygen content (Figure 2e).

### 3.2. Analysis of Clogging in the Submerged Entrance Nozzle

**Figure 3:**
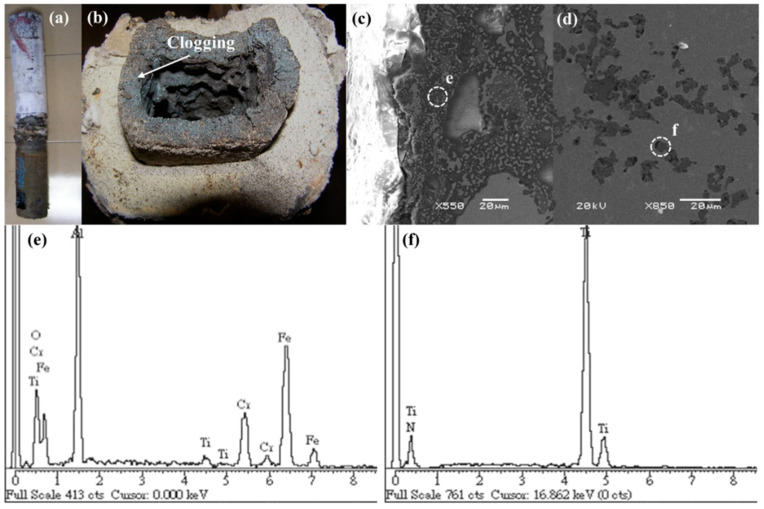
Photographs of (**a**) the submerged entrance nozzle and (**b**) clogging in the submerged entrance nozzle, SEM images of (**c**) the clogging near the inner wall of the outlet of the submerged entrance nozzle and (**d**) the inner wall of the submerged entrance nozzle, and EDS profiles of (**e**) position “e” in (**c**) and (**f**) position “f” in (**d**).

Figure 3a shows that under casting conditions which include a 30 °C overheat and a casting speed of 1.2 m/min, the nozzle was severely clogged, with a clog thickness of approximately 20 mm (Figure 3b). Further observation of the clog using SEM revealed the presence of dendritic inclusions near the inner wall of the submerged entrance nozzle, as is shown in Figure 3c. The EDS results in Figure 3e show high titanium, aluminum, and oxygen content in the dendritic inclusions (marked as “e” in Figure 3c). In addition, diamond-shaped inclusions were observed on the inner wall of the submerged entrance nozzle, as is shown in Figure 3e, and they contained high amounts of titanium and nitrogen, as is shown in Figure 3f.

**Figure 4:**
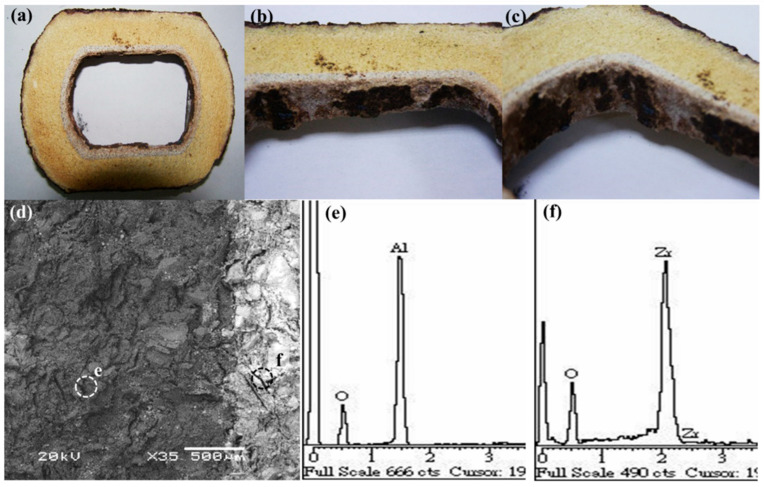
Photographs of (**a**) the outlet of the steel nozzle after casting and (**b**,**c**) the inner wall of the steel nozzle, (**d**) SEM image of the steel nozzle, and EDS spectra of (**e**) position “e” in (**d**) and (**f**) position “f” in (**d**).

Furthermore, the inner wall of the steel nozzle was examined after the clog was removed (Figure 4a–c). This wall was non-uniform (Figure 4a–c), which suggested that the removal of the clog damaged the steel nozzle. Figure 4d shows an SEM image of the inner wall of the steel nozzle. The area on the right of Figure 4d shows the location of the clog. The EDS results, which are shown in Figure 4d, indicate that site “e” mainly comprises aluminum and oxygen, suggesting the presence of an aluminum oxide refractory material. The area from which the clog was removed from the steel nozzle (site “f” in Figure 4d) contained high amounts of zirconium and oxygen (Figure 4f).

### 3.3. Mechanism of Surface Peeling

The surface peeling of the TCFSS strips is attributed to the addition of titanium, which tends to combine with oxygen in molten steel to form titanium oxide. The formation of titanium oxide induces rapid conduction in molten steel owing to the small wetting angle between titanium oxide and molten steel [11]. Consequently, the temperature of the molten steel that is in contact with the inner wall of the nozzle decreases rapidly, and this generates oxide inclusions in the solidified alloy and forms a dendritic layer in the nozzle.

**Figure 5:**
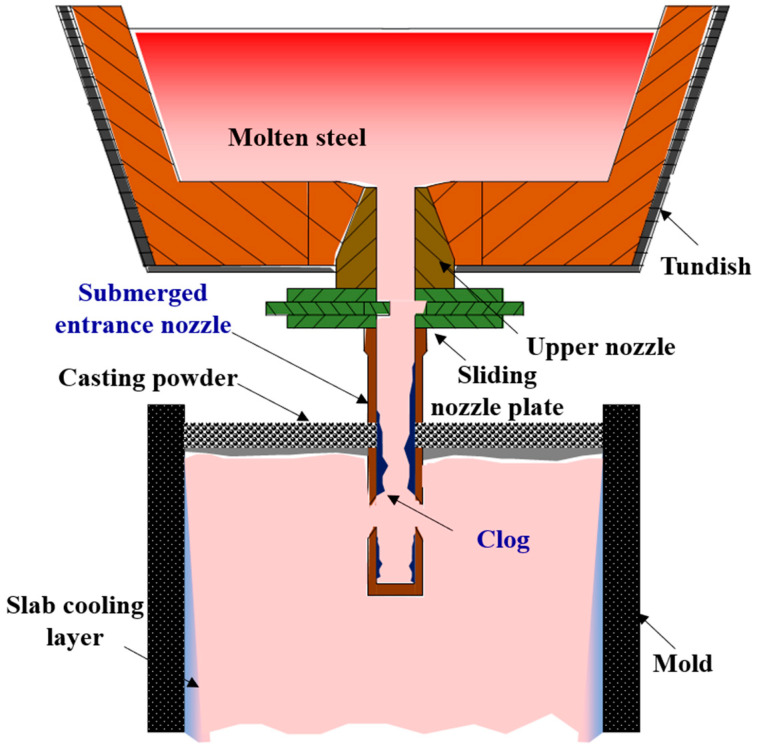
Schematic of the clog formation on the inner wall of the outlet of the submerged entrance nozzle.

The thickness of the dendritic layer eventually increases, thereby clogging the inner wall of the outlet of the steel nozzle, as is shown in Figure 5. Titanium and aluminum oxides also form inclusions in slabs when molten steel solidifies [17]. Owing to the low ductility of titanium and aluminum oxide inclusions, interlayer defects form around these inclusions during hot rolling. In contrast, titanium nitride, which has high hardness, is embedded into the substrate without deformation and the diamond shape of titanium nitride is maintained, as is shown in Figure 2c. Finally, after annealing, surface pickling occurs, and surface peeling occurs on the TCFSS strips when they are subjected to winding tension.

### 3.4. Analysis of the Steel Nozzle Outlet for Different Parameters

**Figure 6:**
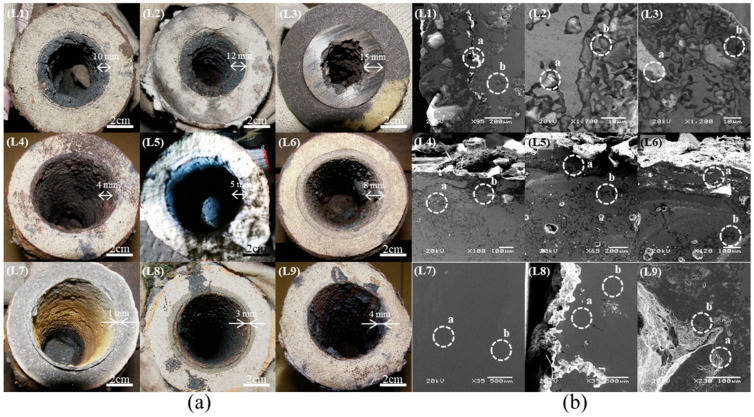
(**a**) Photographs and (**b**) SEM images of steel nozzles L1–L9. The composition of sites a and b in (**b**) was shown in Table 2.

Figure 6a shows photographs and corresponding SEM images of the steel nozzle outlets of samples L1–L9 (Figure 6b). With the exception of L7, all of the nozzles were highly clogged (3–15 mm), as is shown in Figure 6a. As Table 2 shows, the clogging in samples L1–L7 (Figure 6b) contained elements such as titanium, aluminum, nitrogen, and oxygen, whereas those in samples L8 and L9 contained only iron and chromium.

**Figure 7:**
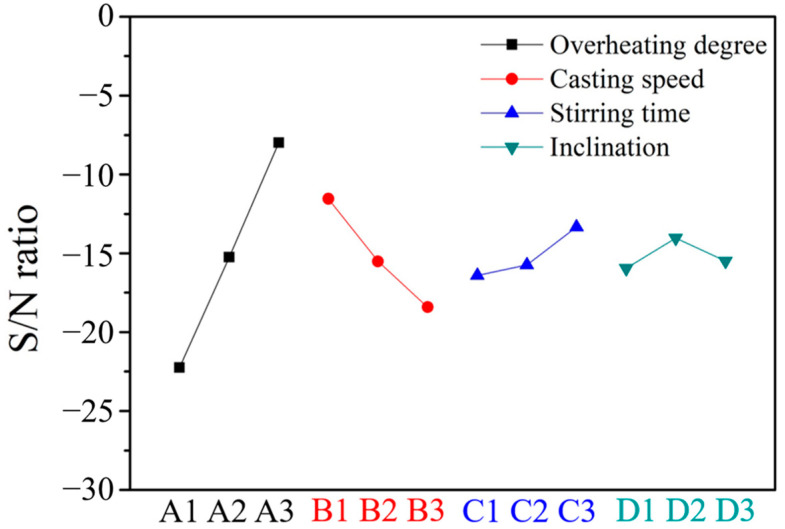
Main effect plot of the S/N ratios at various clog thicknesses.

The clog thicknesses and the calculated S/N ratios obtained from the response graphs plotted in Figure 7 are listed in Table 3. The contributions of the four factors, namely, factors A (degree of overheating), B (casting speed), C (stirring time), and D (inclination), were 76.6%, 17.9%, 1.5%, and 3.9%, respectively (Table 4). The results indicated that the order of significance of the four factors was A > B > D > C. The relatively small effect of the stirring time was a result of its level being set within a small range (10 s, 11 s, and 12 s). Stirring with argon gas causes the oxides to float on molten steel. Stirring for a long time decreases the temperature of the molten steel, and therefore stirring was conducted for only a short time.

### 3.5. Analysis of the Surface Peeling Defects of the TCFSS Strips Produced Using Different Parameters

**Figure 8:**
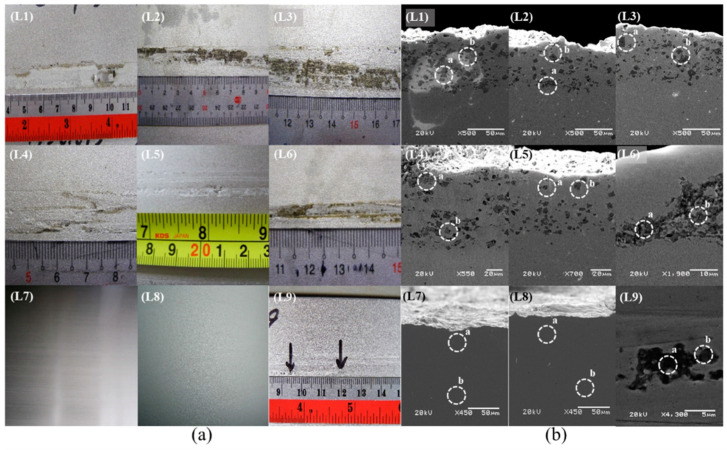
(**a**) Photographs and (**b**) cross-sectional SEM images of samples L1–L9. The composition of sites a and b in (**b**) was shown in Table 5.

Figure 8 shows photographs and corresponding SEM images of samples L1–L9. Figure 8a reveals that, with the exceptions of samples L7 and L8, surface peeling was evident in all the samples. A maximum peeling width of 23 mm was observed for sample L3, and a minimum peeling width of 4.5 mm was observed for sample L9. Numerous black spots close to the surface in samples L1–L6 and L9 can be observed in the cross-sectional SEM images (Figure 8b). However, no spots were observed in samples L7 and L8. These black spots were mainly composed of titanium, aluminum, nitrogen, and oxygen, as is summarized in Table 5. However, samples L7 and L8 contained only iron and chromium, which are the main components of stainless steel.

**Figure 9:**
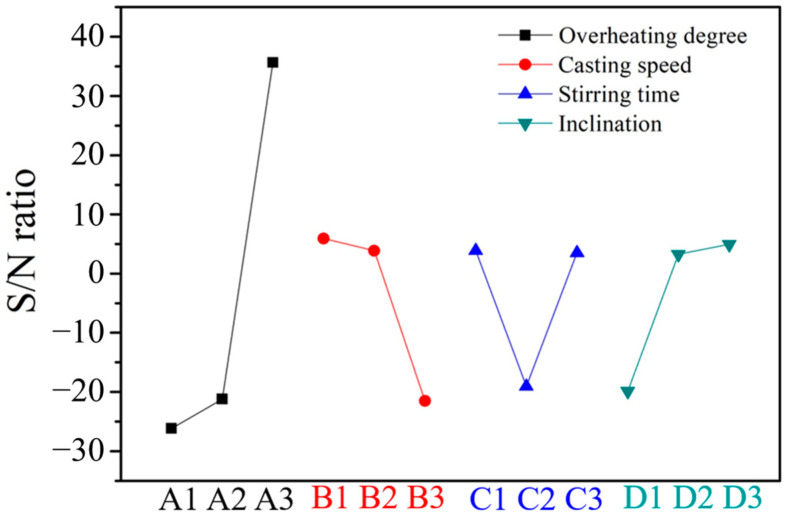
Main effect plot of S/N ratios at various peeling widths.

Table 6 summarizes the response graphs of the peeling widths and the calculated S/N ratios plotted in Figure 9. As Table 7 shows, the contributions of factors A, B, C, and D are 66.4%, 13.1%, 10.8%, and 9.7%, respectively. The results indicated that the general order of significance of the four factors was A > B > C > D.

### 3.6. Relationship between the Casting Parameters and the Surface Quality of the TCFSS Strips

**Figure 10:**
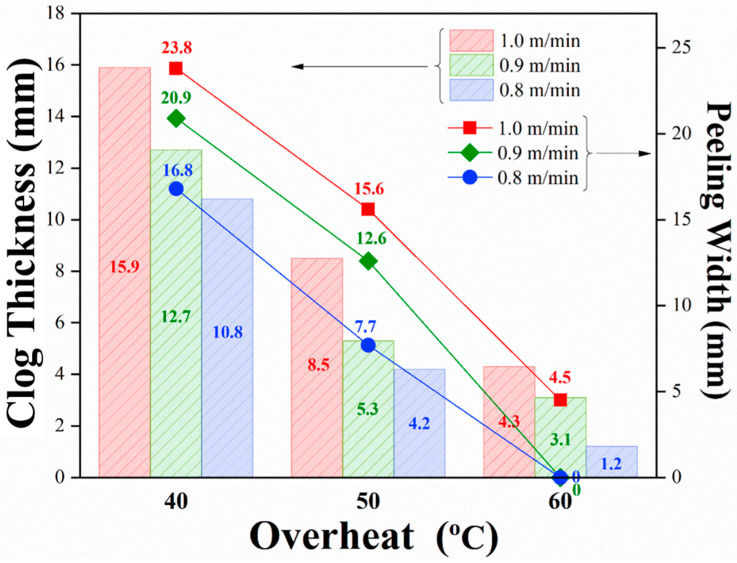
Relationship between peeling defect size and process parameters.

The relationship between the clog thickness at the outlet of the steel nozzle and the casting parameters is shown in Figure 10. The results showed that the clog thickness decreased as the degree of overheating increased and increased as the casting speed increased. In addition, as is shown in Figure 10, the peeling width of the TCFSS strip decreased as the degree of overheating increased and increased as the casting speed increased. This is because the increased degree of overheating [12] increased the fluidity of the molten steel [18]. Furthermore, a decrease in the casting speed results in the adequate floating of titanium oxide, aluminum oxide, and titanium nitride [19], which effectively reduces clogging in the steel nozzle and inhibits the formation of peeling defects on the surface of the cold-rolled steel strip.

### 3.7. Optimal Parameters

**Figure 11:**
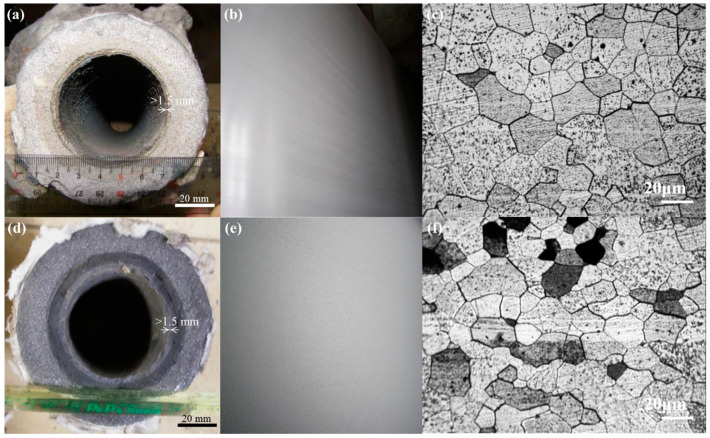
Photographs of (**a**) the outlet of the steel nozzle and (**b**) sample A3B1C3D2 (L7), (**c**) an optical microscope image of A3B1C3D2 (L7), photographs of (**d**) the outlet of the steel nozzle and (**e**) sample A3B1C1D3, and (**f**) an optical microscope image of sample A3B1C1D3.

The optimal clog thickness was approximately 1.2 mm, which was formed as a result of the parameter combination A3B1C3D2 (L7), i.e., a degree of overheating of 60 °C, a casting speed of 0.80 m/min, a stirring time of 12.0 s, and an inclination angle of 6.0°. The optimum peeling width was 0 mm (no peeling), which was achieved using the parameter combination A3B1C1D3, i.e., a degree of overheating of 60 °C, a casting speed of 0.80 m/min, a stirring time of 10.0 s, and an inclination angle of 6.2°. Notably, samples A3B1C3D2 (L7) and A3B1C1D3 also exhibited good surface quality and no peeling defects (Figure 7a). The results of the experiments are shown in Table 8 and Table 9 and in Figure 11. The compositions and mechanical properties of the A3B1C3D2 (L7) and A3B1C1D3 samples are listed in Table 8 and Table 9, respectively. The properties of samples A3B1C3D2 (L7) and A3B1C1D3 fulfilled the design requirements. Figure 11a,d show photographs of the outlets of the steel nozzles of A3B1C3D2 (L7) and A3B1C1D3. Both samples experienced thin clogging of <1.5 mm. No peeling was observed on the sample surfaces of A3B1C3D2 (Figure 11b) or A3B1C1D3 (Figure 11e). In addition, samples A3B1C3D2 (L7) (Figure 11c) and A3B1C1D3 (Figure 11f) are ferrites (*α*-Fe), and the number of grain sizes is in the range of 3–4.

Based on the experimental results and analysis, it was concluded that the degree of overheating and the casting speed have the most influence on the clog thickness and peeling width. In addition, these two factors interacted with the casting behavior. Stolyarov et al. [20] found that casting overheating temperatures over 30 °C and withdrawal speeds of less than 2.5 m/min improved the purity of cast billets. The casting velocity is a necessary complement to the degree of overheating in decreasing the shrinkage cavity size. For instance, a high degree of overheating results in a large solidifying contraction volume, which can be compensated for by slow casting [21].

## 4. Conclusions

This study utilizes the Taguchi method for the optimization of casting parameters to prevent surface peeling defects, which are caused by clogging owing to titanium and aluminum oxides that are aggregated in the nozzle during the casting of TCFSS manufactured from scrap. Based on the experimental results and analysis, it can be concluded that the degree of overheating has the greatest effect on the clog thickness, followed by casting speed, inclination angle, and stirring time. The reduction in the clog thickness to a value less than 1.5 mm was observed in sample A3B1C3D2 owing to the following optimal parameters: a degree of overheating of 60 °C, a casting speed of 0.80 m/min, a stirring time of 12.0 s, and an inclination angle of 6.0°. The optimal parameters to prevent peeling defects corresponded to sample A3B1C1D3, i.e., a degree of overheating of 60 °C, a casting speed of 0.80 m/min, a stirring time of 10.0 s, and an inclination angle of 6.2°. The results obtained using the Taguchi optimization method revealed that no peeling defects were present on the surface of the TCFSS strip when the casting overheat was fixed at 60 °C with a casting speed of 0.8–0.9 m/min. The TCFSS strips produced using the optimized parameters exhibited the required mechanical properties (i.e., a tensile strength of ≥415 MPa, yield strength of ≥205 MPa, elongation of ≥22%, and hardness of ≤89 HRB) and satisfied the design criteria.

## Figures and Tables

**Table 1:** Levels according to the L9 orthogonal array.

	A	B	C	D
Experiment Number	Degree of Overheating (°C)	Casting Speed (m/min)	Stirring Time (s)	Inclination (°)
1	40	0.8	10.0	5.8
2	40	0.9	11.0	6.0
3	40	1.0	12.0	6.2
4	50	0.8	11.0	6.2
5	50	0.9	12.0	5.8
6	50	1.0	10.0	6.0
7	60	0.8	12.0	6.0
8	60	0.9	10.0	6.2
9	60	1.0	11.0	5.8

**Table 2:** EDS results obtained from the clogs in steel nozzles L1–L9 (the sites detected are shown in Figure 6b).

Experiment Number		Element (at %)						
		Al	Cr	Ti	Ca	N	O	Fe
L1	Site a	33.64	-	11.39	4.56	-	50.40	0
	Site b	-	12.39	30.23	-	9.67	-	47.71
L2	Site a	27.86	12.06	1.31	-	-	17.27	41.50
	Site b	-	12.40	36.44	-	10.91	-	40.25
L3	Site a	41.90	4.43	1.70	-	-	4.55	47.42
	Site b	-	-	79.51	-	20.49	-	0
L4	Site a	-	-	5.16	4.88	-	46.76	43.20
	Site b	1.20	6.64	42.63	-	7.80	23.41	18.32
L5	Site a	27.86	12.06	1.31	-	-	17.27	41.50
	Site b	-	3.59	70.63	-	11.47	-	14.31
L6	Site a	40.87	-	5.19	3.36	-	42.67	7.91
	Site b	-	5.74	57.24	-	20.60	-	16.42
L7	Site a	-	18.73	-	-	-	-	81.27
	Site b	-	18.50	-	-	-	-	81.50
L8	Site a	-	18.79	-	-	-	-	81.21
	Site b	-	18.62	-	-	-	-	81.38
L9	Site a	1.13	14.59	9.77	-		5.05	69.64
	Site b	-	-	76.62	-	14.04	-	9.34

**Table 3:** Individual clog thicknesses, average thicknesses, standard deviations, and S/N ratios of samples L1–L9.

Experiment Number	Sample			Average Value	Standard Deviation	S/N
	1	2	3			
L1	10.4	10.8	11.2	10.8	0.4	−20.67
L2	12.7	12.3	13.0	12.7	0.4	−22.06
L3	15.5	16.2	15.9	15.9	0.4	−24.01
L4	4.3	4.0	4.4	4.2	0.2	−12.54
L5	5.3	5.8	4.9	5.3	0.5	−14.57
L6	8.5	8.8	8.3	8.5	0.3	−18.63
L7	1.0	1.3	1.2	1.2	0.2	−1.41
L8	3.1	3.1	3.2	3.1	0.1	−9.92
L9	4.4	4.0	4.4	4.3	0.2	−12.61
Average value				7.3	0.3	−15.16

**Table 4:** ANOVA results at various clog thicknesses.

Factor	SS	DOF	Var	Probability	Contribution
A	305.26	2	152.628	0.00	76.61%
B	71.45	2	35.723	0.13	17.93%
C	15.70	2	7.849	0.61	3.94%
D	6.04	2	3.019	0.82	1.52%
Error	1.60				0.40%
Total	400.05	8			100.00%

**Table 5:** EDS results for samples L1–L9 (the sites detected are shown in Figure 8b).

Experiment Number		Element (at %)						
		Al	Cr	Ti	Ca	N	O	Fe
L1	Site a	4.73	24.79	2.78	1.08	-	37.61	29.10
	Site b	-	-	79.23	-	20.77	-	0
L2	Site a	-	10.20	3.95	3.92	-	27.44	54.49
	Site b	2.52	-	71.91	-	25.57	-	0
L3	Site a	1.34	0.94	59.59	-	-	34.51	3.62
	Site b	-	6.25	65.80	-	18.52	-	9.43
L4	Site a	6.45	12.12	2.89	2.26	-	31.36	44.92
	Site b	-	5.21	63.40	-	14.91	-	16.48
L5	Site a	2.27	9.18	3.17	4.09	-	31.01	50.28
	Site b	-	-	83.75	-	16.25	-	0
L6	Site a	13.91	15.02	6.87	5.44	-	39.14	19.62
	Site b	-	83.59	-	-	16.41	-	0
L7	Site a	-	17.88	-	-	-	-	82.12
	Site b	-	17.90	-	-	-	-	82.10
L8	Site a	-	17.79	-	-	-	-	82.21
	Site b	-	17.93	-	-	-	-	82.07
L9	Site a	1.91	40.31	1.92	1.11	-	10.75	44.00
	Site b	-	5.14	68.16	-	12.75	-	13.95

**Table 6:** Individual peeling widths, average peeling widths, standard deviations, and S/N ratios of samples L1–L9.

Experiment Number	Sample			Average Value	Standard Deviation	S/N
	1	2	3			
L1	17.0	17.0	16.5	16.8	0.3	−24.52
L2	21.3	20.7	20.6	20.9	0.4	−26.39
L3	23.4	23.8	24.1	23.8	0.4	−27.52
L4	8.0	7.2	7.8	7.7	0.4	−17.70
L5	12.2	12.5	13.0	12.6	0.4	−21.99
L6	15.6	15.6	15.7	15.6	0.1	−23.88
L7	0.001	0.001	0.001	0.0	0.0	60.00
L8	0.001	0.001	0.001	0.0	0.0	60.00
L9	4.8	4.2	4.5	4.5	0.3	−13.08
Average value				11.32	0.2	−3.90

**Table 7:** ANOVA results at various peeling widths.

Factor	SS	DOF	Var	Probability	Contribution
A	7071.50	2	3535.750	0.00	66.36%
B	1399.53	2	699.767	0.21	13.13%
C	1034.46	2	517.231	0.31	9.71%
D	1151.41	2	575.703	0.27	10.80%
Error	1.56				0.01%
Total	10,658.46	8			100.00%

**Table 8:** Compositions of samples A3B1C3D2 (L7) and A3B1C1D3.

Experiment Number	Element (at %)							
	C	Si	Al	Cr	Ti	Ca	N	Ref.
Ingredient design	≤0.020	0.20~ 0.50	≤0.10	17.00~ 18.00	0.35~ 0.50	-	≤0.010	[22]
A3B1C3D2 (L7)	0.015	0.49	0.03	17.65	0.47	0.002	0.008	
A3B1C1D3	0.014	0.42	0.03	17.58	0.46	0.002	0.007	

**Table 9:** Mechanical properties of samples A3B1C3D2 (L7) and A3B1C1D3.

Experiment Number	Tensile Strength (MPa)	Yield Strength (MPa)	Elongation (%)	Hardness (HRB)	Grain Number	Ref.
Mechanical design	≥415	≥205	≥22	≥89	-	
AISI430SS	480	340	27.5			[23]
A3B1C3D2 (L7)	483	320	30	78	4	
A3B1C1D3	435	308	33	75	3	

## Data Availability

Data sharing is not applicable.
